# Generation of transgenic chickens by the non-viral, cell-based method: effectiveness of some elements of this strategy

**DOI:** 10.1007/s13353-018-0429-6

**Published:** 2018-01-25

**Authors:** Marek Bednarczyk, Izabela Kozłowska, Paweł Łakota, Agata Szczerba, Katarzyna Stadnicka, Takashi Kuwana

**Affiliations:** Department of Animal Biochemistry and Biotechnology, University of Science and Technology, Bydgoszcz, Poland

**Keywords:** Chicken, Transgenesis, Non-viral strategy

## Abstract

Transgenic chickens have, in general, been produced by two different procedures. The first procedure is based on viral transfection systems. The second procedure, the non-viral method, is based on genetically modified embryonic cells transferred directly into the recipient embryo. In this review, we analyzed the effectiveness of important elements of the non-viral, cell-based strategy of transgenic chicken production. The main elements of this strategy are: isolation and cultivation of donor embryonic cells; transgene construction; cell transfection in vitro; and chimera production: injection of cells into recipient embryos, raising and identification of germline chimeras, mating germline chimeras, transgene inheritance, and transgene expression. In this overview, recent progress and important limitations in the development of transgenic chickens are presented.

## Introduction

Various methods have been developed to produce successfully transgenic animals, including: direct DNA microinjection into the pronucleus (Brinster et al. 1981); viral vector transfer (Salter et al. 1987); injection of in vitro transfected embryonic stem cells into the blastocyst (Stewart et al. 1985); and cloning of transfected nuclei transferred into enucleated oocytes (Keefer et al. 2001). However, the production of transgenic birds has been hampered by the yolk-laden structure of the ovum and their unique reproductive system. Direct DNA microinjection, a frequently applied technique in mammals, is almost impossible in birds, because fertilization in the hen occurs in the infundibulum of the reproductive tract and fertilization in birds is polyspermic (Perry 1987). Also, identification of the male pronucleus among the supernumerary spermatozoa is difficult, as is the return of the ovum to the oviduct of a fistulated hen (Pancer et al. 1989). Consequently, transgenic chickens have, in general, been produced by two different procedures (reviewed by Song et al. 2010). The first procedure is based on viral transfection systems. The second procedure, the non-viral method, is based on genetically modified embryonic cells transferred directly into the recipient embryo.

Although viral transfection systems allow for efficient introduction and expression of transgenes in chicken dividing and non-dividing cells (McGrew et al. 2004), as well as in the chicken oviduct (Liu et al. 2015; Wu et al. 2015), there are some important limitations to this method. The lentiviral vectors have three main limitations (reviewed by Bednarczyk 2016): (i) restriction of the size of the vector genome to less than 8 to 10 kb (Wu et al. 2010); however, the promoters necessary to drive specific expression in some cells exceed this size; (ii) vector insertion can cause disruption of endogenous genes by insertional mutagenesis or the transactivation of neighboring endogenous genes (Li and Lu 2010); and (iii) integrated lentiviral vectors are subject to positional effects (Yi et al. 2011). Moreover, the viral transduction technique is characterized by high embryonic lethality rates, and relatively low and unpredictable rates of germline transmission and production of transgenic chickens (McGrew et al. 2004; Park and Han 2012b). However, a much more important limitation is public concern, which has questioned the safety of lentivirus-based technology. The fact that the lentiviral vector is derived from lentiviruses, which cause chronic, sometimes life-threatening, illnesses in humans and animals, is a strong argument.

Therefore, over the last few years, some alternative strategies have been developed and the idea of the generation of transgenic chickens through chimeric intermediates described (Petitte et al. 1990). The generation of transgenic chickens has been attempted through chimeric intermediates produced by the transfer of blastodermal cells. The same idea has been implemented in many other experiments, for example, Vick et al. (1993); however, in this instance, primordial germ cells (PGC) were proposed as the vehicle for the introduction of the transgene into the chicken genome. Elaboration of the extended culture method for those cells (van de Lavoir et al. 2006) and the development of assays of their genetic modification (Macdonald et al. 2012; Park and Han 2012a) have culminated in the production of transgenic or chimeric chickens expressing important therapeutic proteins in the oviduct (Lillico et al. 2007; Park et al. 2015). A hen which can secrete a lot of protein in its oviduct and which can regularly, in a 20–24-h cycle, produce eggs is a very attractive vehicle for the recovery of therapeutic proteins, because the sterile contents of the eggs are enveloped in a hard shell. Subsequently, the first therapeutic protein, recombinant human lysosomal acid lipase, produced in the oviduct of a genetically engineered White Leghorn hen was commercialized by Alexion Pharmaceuticals, USA in 2015.

However, the effectiveness of the transgenic method in birds is still very low in many cases. In this review, we analyzed the effectiveness of important elements of the non-viral, cell-based strategy of transgenic chicken production from the literature cited in the Web of Science database, and the experiments that have been carried out in our laboratory. The main elements of the non-viral, cell-based strategy are:

- Isolation and cultivation of donor embryonic cells;
- Transgene construction;
- Cell transfection in vitro; and
- Chimera production: injection of cells into recipient embryos, raising and identification of germline chimeras, mating germline chimeras, transgene inheritance, and transgene expression.

## Isolation and cultivation of donor embryonic cells

In the non-viral cell-based method, PGC have usually been used to create the transgenic chickens. In contrast to mammals, avian PGC use the circulatory system of a forming embryo to move from the region of the germinal crescent to the future gonads. The unique migratory properties of PGC provide opportunities for the identification/isolation of these cells from: the blastoderm at stage X (Eyal-Giladi and Kochav 1976); blood of 2.5–3-day-old embryos (stages 13–17 of Hamburger and Hamilton 1951; and from the gonads of 5–7-day-old embryos (stages 26–31) (reviewed by Chojnacka-Puchta et al. 2012).

PGC can be identified based on certain morphological features, such as: presence of a large spherical nucleus; very well developed Golgi apparatus and endoplasmic reticulum (Fujimoto et al. 1976); and presence of refractive lipids in cytoplasm and numerous grain reserve substances (Zhao and Kuwana 2003). In addition, to identify PGC, the following methods are used: periodic acid–Schiff (PAS) staining (Fujimoto et al. 1976) and immunological markers against cell surface glycoproteins present in PGC, such as SSEA-1 (stage-specific embryonic antigen 1) or CVH (chicken vasa homolog) proteins located in their cytoplasm (reviewed by Nakamura et al. 2013).

**Fig. 1:**
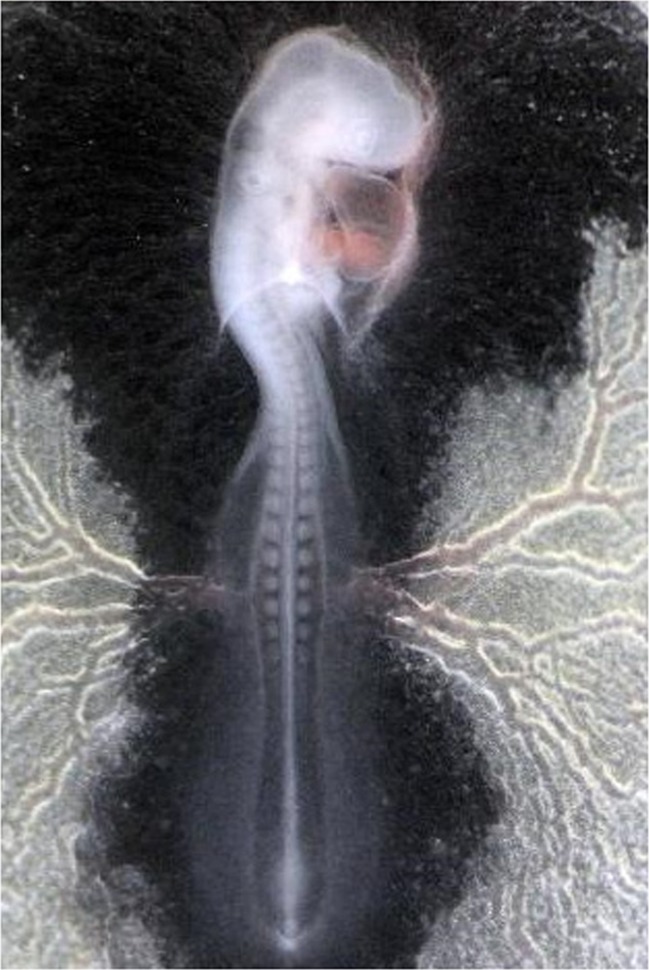
Chicken embryo at stage 14 with 22 somites (50–53 h of incubation), magnification ×16

Due to the small number of PGC isolated from stage X embryos, this source of cells is not very popular. Blood at stages 13–17 becomes a potential source of PGC for the production of chicken chimeras. In particular, blood coming from stage 14 (Fig. 1) appears to be the most suitable for this purpose, as the concentration of circulating PGC is then the highest (Tajima et al. 1999).

PGC have been isolated by different purification techniques, including Ficoll, Nycodenz, Percoll density gradient centrifugation, ammonium–chloride–potassium (ACK) lysis buffer, immunomagnetic separation (IMS), and fluorescence-activated cell sorting (FACS) (Chojnacka-Puchta et al. 2015). However, the method of PGC isolation from the embryonic blood was reported to be complicated and resulted in a very small number of PGC being obtained (approximately 0.048% of the total cells in blood) (Yasuda et al. 1992), which limits the use of this method of isolation for more technically advanced transgenic manipulation.

The most practical method of obtaining PGC seems to be their isolation from the gonads of 5–7-day-old embryos. For this purpose, the gonads are fragmented and/or digested using 0.25% trypsin–0.01% EDTA in PBS[−] solution. Compared to the other two methods, the number of PGC per embryo obtained by this method is the highest, thus increasing the success of chimera generation. Nakajima et al. (2011) suggested a new and simple method of collecting highly purified (about 50%) gonadal PGC by the incubation of gonads (at a temperature of 37.8 °C and 5% CO_2_) from 7-day-old embryos in PBS solution deprived of calcium and magnesium ions (PBS[−]). At present, this is the most effective method of PGC isolation from the gonads of chicken embryos, because it allows, in a short time, the collection of a large number of highly purified and viable PGC (Nakamura et al. 2013).

**Fig. 2:**
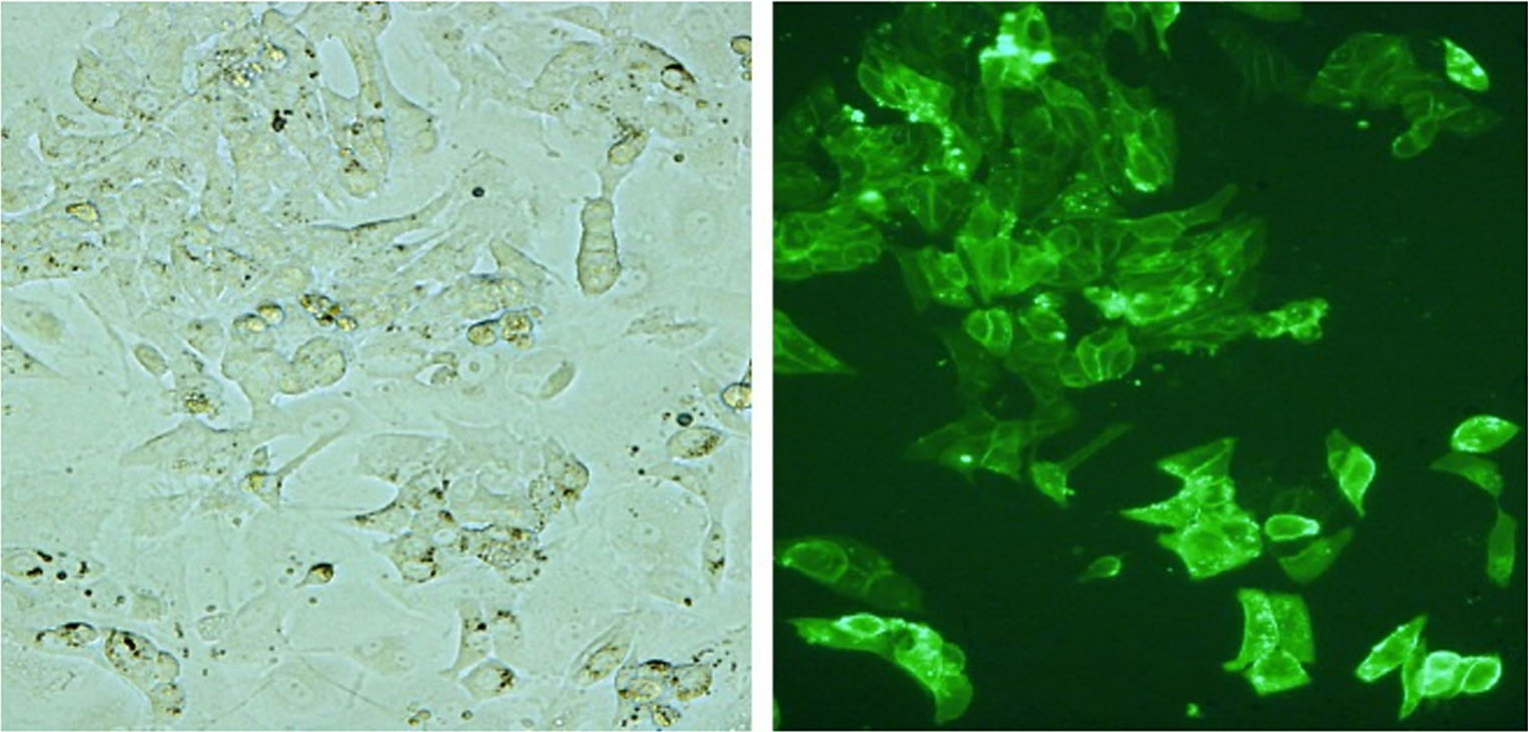
Cultured primordial germ cells (PGC) on feeder cells. PGC proliferated and formed small colonies on the feeder cells after 14 days’ cultivation (*left*) and were SSEA-1 (stage-specific embryonic antigen 1) positive (*right*)

However, the number of PGC that can be obtained from one embryo is limited (Tajima et al. 1999), regardless of the method of their isolation from the blastoderm, from the blood, or from the gonads. To make effective use of the small number of PGC, it is essential to increase the number by culturing cells in vitro (Fig. 2).

Several attempts have been made to maintain and propagate avian PGC in vitro. The first successful long-term culture of PGC was reported by van de Lavoir et al. (2006). PGC were obtained from embryonic blood and were cultured on mouse STO or buffalo rat liver (BRL) feeder cells by BRL-conditioned KnockOut DMEM with basic fibroblast growth factor (bFGF) and stem cell factor (SCF) supplementation. The PGC were cultured for more than 35 days and successfully migrated to the germinal ridges after transfer into the bloodstream of recipient embryos. These cells also differentiated into functional gametes in recipient gonads. The effectiveness of this culture system for PGC was confirmed by Macdonald et al. (2010) and Miyahara et al. (2014). Choi et al. (2010) developed a feeder-free culture system for PGC and found that bFGF is one of the important factors for the proliferation of PGC and also for maintaining the undifferentiated state of these cells. Furthermore, Park and Han (2012a) modified the culture system for PGC using mouse fibroblast feeder cells and produced germline chimeric chickens with more than a 95% germline transmission rate of donor PGC.

These culture systems use xeno-animal cells as feeder cells, and the use of this system may increase the risk of a cross-transfer of animal pathogens from other animal cells. It is, therefore, recommended to use chicken cells as feeder cells for culturing chicken PGC. However, long-term culture of chicken PGC isolated from embryonic blood using feeder cells derived from chicken embryos has not been successful. The propagation of female PGC in this system is less efficient compared with that of male PGC (van de Lavoir et al. 2006; Macdonald et al. 2010). Similarly, in the method developed by Tonus et al. (2016) that allows culture for more than 1 year, cell proliferation, and cryobanking of primary cultures of PGC, all the resulting lines appeared devoid of female cells, despite having been initially pooled from male and female embryos. In contrast, Naito et al. (2015) reported the successful long-term culture of female PGC in vitro and subsequent generation of viable offspring via germline chimeric chickens. In this method, female PGC are present in mixed-sex PGC populations cultured for more than 90 days. Moreover, optimal culture conditions of female PGC are different from those for male PGC.

As a summary of this part of the review, we can conclude that too little is yet known about the effects of long-term PGC cultivation in vitro on cells’ functional ability. Therefore, further investigations are needed to support the higher efficiency of chicken PGC culture and the suppression of cell differentiation by long-term culture in vitro.

## Transgene expression constructs

According to the recommendations of the Center for Genetic Medicine (http://www.cgm.northwestern.edu/cores/ttml/transgenic-projects/transgenic-construct-design.html), standard transgenic constructs should contain all the 5′ and 3′ regulatory elements necessary for transgene expression, the gene of interest and/or marker gene, and restriction sites that allow isolation of a full-length linear transgenic fragment. The addition of sequences that potentially increase the level of transgene expression should be considered. A strategy for the detection of the transgene or its product (i.e., epitope tags on transgenic protein) should also be inherent to the construct design.

The promoter/enhancer elements driving gene expression are of some importance, depending on whether ubiquitous or tissue-specific expression is desired (Chapman et al. 2005). There are several known ubiquitous promoters which are strongly active in a wide range of cells, tissues, and cell cycles, e.g., chicken β-actin, cytomegalovirus (CMV) enhancer (CCAG or CAG promoter), histone H4 promoter, or phosphoglycerol kinase (PGK) promoter. The cytomegalovirus immediate–early gene promoter/enhancer (CMV) is a highly efficient promoter in many vertebrates, but in chickens, it seems to be less efficient than β-actin (reviewed by Chapman et al. 2005).

A number of genes are expressed only in a specific tissue or in response to unique environmental signals. The possibility of tissue-specific transgene expression opens new avenues for biological research and for genetic manipulation in birds. McGrew et al. (2010) investigated the possibility of utilizing rodent regulatory elements to drive transgene expression in the skeletal muscle of chickens, and they demonstrated that myosin light chain promoters/enhancers (MLC) drive skeletal muscle gene expression in transgenic chickens.

The most demanding, but also most explored, area is expression of the ovalbumin gene. The chicken ovalbumin promoter, which produces yields of ovalbumin that constitute about 54% of egg white total protein, is one of the strongest tissue-specific promoters. This expression mechanism induced by estrogen is only found in tubular gland cells of the hen’s oviduct. The chicken ovalbumin promoter has been used to induce oviduct-specific expression of a therapeutic protein in transgenic chicken bioreactors (Chojnacka-Puchta et al. 2015; Harvey and Ivarie 2003; Kwon et al. 2010; Lillico et al. 2007; Liu et al. 2015; Park et al. 2015). The ovalbumin (Ov) gene contains a number of regulatory elements that control its transcriptional activity and restrict expression to the avian oviduct. Lillico et al. (2007) constructed a synthetic oviduct expression vector with 2.8 kb of the 5′-flanking region on the ovalbumin promoter and estrogen-responsive enhancer (ERE) element. Another study (Zhu et al. 2005) also reported that human monoclonal antibody was controlled by the ovalbumin gene. The vectors contain 7.5- or 15-kb sequences on the 5′-flanking region of the ovalbumin promoter and 15.5 kb on its 3′-flanking region.

**Fig. 3:**
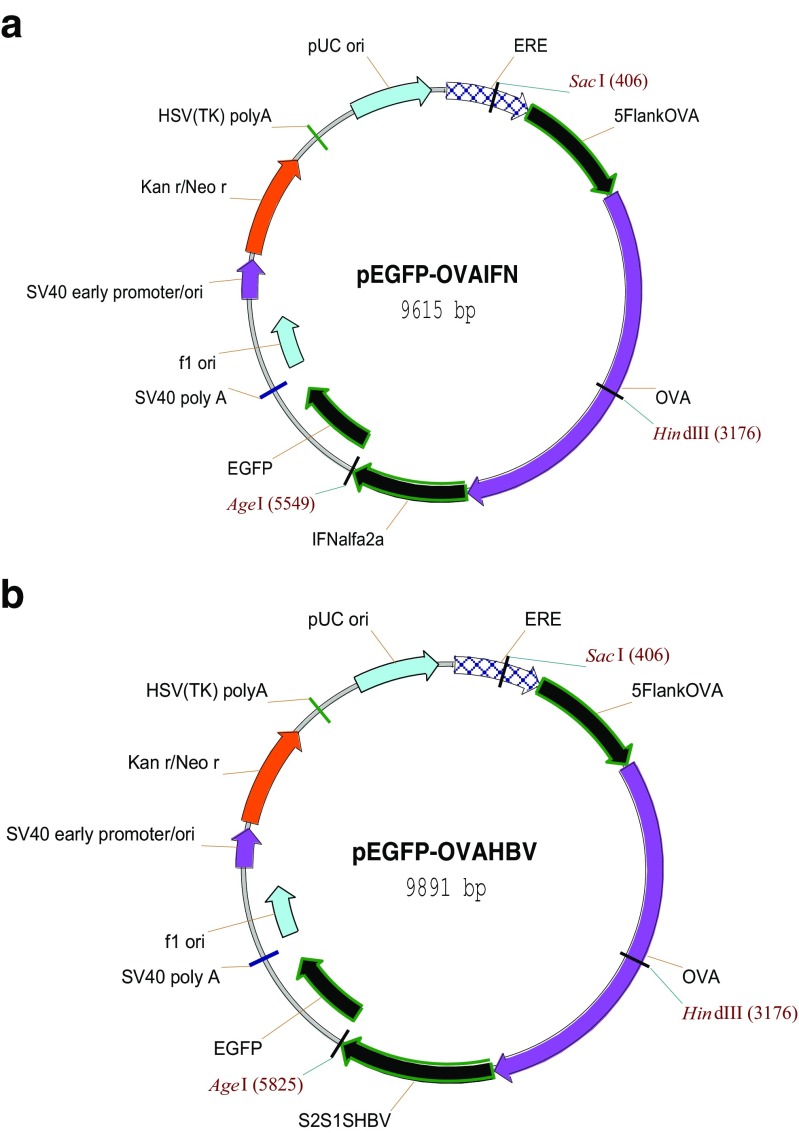
Schemes of the pEGFP-OVAIFN (**a**) and pEGFP-OVAHBV (**b**) expression vectors (Bednarczyk et al. 2003; Chojnacka-Puchta et al. 2015)

In our studies (Bednarczyk et al. 2003; Chojnacka-Puchta et al. 2015) of the expression of the human interferon alpha-2a gene (IFNα2a) or hepatitis B virus surface antigen (HBsAg), the specific vectors were constructed under the control of the chicken ovalbumin promoter (OVA), and by strengthening sequences which included the chicken oviduct-specific and enhancer-like (COSE) region and ERE element (Fig. 3).

However, the specific elements associated with the ovalbumin gene that direct oviduct-specific expression have not yet been fully defined, so it is difficult to identify the optimal expression vector(s) providing efficient expression of therapeutic proteins in the chicken oviduct.

## Cells transfection in vitro

PGC are characterized by their low ability to induce effective and persistent transfection. Additionally, there are several obstacles to this process, because germ cells are relatively transcriptionally quiescent and prone to switching off transgene expression (Seydoux and Braun 2006). In general, the evaluation of PGC transfection efficiency centers on the selection of a suitable method for the genetic modification of cells, as well as its evaluation system. Usually, transfection efficiency is assessed according to the marker gene encoding the fluorescent protein: EGFP–green, EYFP–yellow, mCherry–red, tdTomato–bright red. Sometimes, the *lacZ* gene encoding a beta-galactosidase protein is used; however, an enzymatic or histochemical assay is necessary for its product identification.

Among the non-viral systems for the delivery of a gene into PGC, electroporation (van de Lavoir et al. 2006; Oishi 2010) and lipofection (Naito et al. 1998; Furuta et al. 2010; Kaleri et al. 2011) techniques have been used to transfer DNA. The main parameters that affect electroporation effectiveness are: pulse amplitude, pulse duration, number of delivered pulses, osmotic pressure (Kotnik et al. 2003). In lipofection, DNA is shielded and packaged into several different natural or synthetic compounds (carriers) to facilitate cellular uptake and intracellular release (reviewed by Grigsby and Leong 2010). These vectors try to mimic viral vectors in terms of assembly and cellular delivery, but have many advantages over viral vectors, such as their easy large-scale production, large transgene capacity, safety, and simplicity.

The transfection efficiency of PGC by synthetic DNA carriers is usually low and transgenes are gradually lost during embryonic development (Naito et al. 1998). After 17 days of incubation following the PGC injection, the *lacZ* gene is detected in only 14.3% (3/21) of embryos examined. Although the transgene (*lacZ* gene) has been detected in the gonads of two hatched chicks (11.1%), it has not been detected in the gonads of chimeric chickens at sexual maturity (Naito et al. 1998). However, successful transfer of exogenous genes into chicken PGC has been achieved by lipofection when the gene was introduced into chickens at stage X of development (Furuta et al. 2010).

There are, however, relatively few studies comparing both methods. Hong et al. (1998) compared two methods for PGC transfection. Electroporation was reported to have an 80% efficiency of DNA transfer, whereas transfection with DNA–liposome complexes was only 17% efficient. Our earlier in vitro and in vivo study (Chojnacka-Puchta et al. 2015) aimed to compare the influences of different chicken PGC (isolated from circulating blood or gonads) purification (ACK, Percoll, or trypsin) and transfection methods (electroporation or lipofection) on the expression of transgenes in vitro and the migration of modified donor cells to the recipient gonads. These data confirmed that the combination of PGC purification methods and transfection methods could be an effective strategy for producing transgenic chickens. The highest average frequency of transgene-transfected PGC (75.8%) was achieved with Percoll density gradient centrifugation and electroporation.

Similarly, for human embryonic stem cells, synthetic DNA carriers (lipofectamine) have been considered a successful approach to transient and stable cell line generation; however, the efficiency of this method appeared to be much lower than that of electroporation (Tabar et al. 2015).

Some authors (Macdonald et al. 2012; Park and Han 2012a) have recently proposed the use of transposon elements such as *piggyBac*, Tol2, and Sleeping Beauty to create a more versatile method to target chicken germline stem cells. Transposons are genetic elements that can relocate between different genomic sites, and the enzyme transposase can excise unique DNA sites and recombine transposons into targeted sites in the genome (Park and Han 2012b). The use of transposon vectors will greatly increase the efficiency of stable genetic modification of PGC (Macdonald et al. 2012). However, it will be necessary to analyze this method further and to explain, for example, the stable expression of the green fluorescent protein (GFP) transgene in multiple tissue types, including heart, brain, liver, intestine, kidney, and gonad, without tissue-specific transgene silencing (Park and Han 2012a). Surprising results have also been derived from an analysis of the progeny of a germline chimera rooster, where only a small number of germ cell-derived offspring were noted: 1 of a total of 518 (Macdonald et al. 2012).

Hitherto, a number of expression vectors have been proposed and numerous techniques have been established for the creation of transgenic birds. To achieve this objective, a reliable in vitro assay system which would serve to verify the efficiency of recombinant gene expression in the oviduct is necessary. The traditional method whereby the transgenic vectors were roughly introduced into the host genome and the tissue-specific protein expression in the egg white from transgenic birds was quantified is both costly and inefficient, because of the lack of vector verification in the target organ, such as the oviduct (Jung et al. 2011). One promising model developed by our group (Kasperczyk et al. 2012) could be the culture of chicken oviduct epithelial cells (COEC) that can be easily transfected with constructs based on oviduct-specific regulatory sequences, for example those derived from the ovalbumin or lysozyme genes. This unique, in-house-developed in vitro culture system for COEC has the potential to produce therapeutic proteins in vitro which would allow the verification of their functions and activity and, most importantly, of the efficiency of recombinant gene expression in the tubular gland cells of the oviduct (Stadnicka et al. 2016).

## Chimera production

The formation of chicken chimera involves PGC isolation from donor embryos, cell transplantation into recipient embryos, and the subsequent regular procedure of embryo development. Petitte et al. (1990) were the first to produce a chicken germline chimera by separating blastodermal cells (containing presumptive PGC) from stage X embryos, and these were then injected into the subgerminal cavity of recipient embryos at the same stage of embryonic development. The resulting rooster had functional sperm; however, a low percentage (1.9%) of germline chimeric chicken was observed. In our experiment (Bednarczyk et al. 2002), blastoderm cells from chicken embryos of a donor breed (Green-legged Partridge-like) were transferred to embryos of a recipient breed (White Leghorn), and among 20 putative chimeric chickens, 6 (30%) produced recipient-derived and donor-derived offspring, indicating that they were germline chimeras.

PGC isolated from the blood of 2.5–3-day-old (stages 13–17) embryos, or the gonads of 5.5–6-day-old (stages 26–28) embryos, can be injected through a window opened in the eggshell into the dorsal aorta of recipient embryos. After injection, the eggshell can be sealed and the egg returned to the incubator and incubated until hatching. Sometimes, the recipient embryos are cultured in host eggshells, as described by Naito et al. (1991), to allow precise manipulation. However, the method of direct cell injection and embryo incubation in sealed eggshells (Chojnacka-Puchta et al. 2015) is considerably easier and more efficient than the transfer of embryos to surrogate eggshells, as was used by Naito et al. (1991).

The production of germline chimeras involves the incorporation of exogenous PGC into the endogenous gonadal tissue of recipient embryos. Therefore, there is a specific competition between the two populations of PGC, endogenous and exogenous, which leads to the production of two types of germ cells: those derived from a donor and those from a recipient. Thus, the proportion of exogenous gametes is determined by the ratio of the number of germ cells of the host to the cells artificially introduced into the gonads of recipient embryos (Nakamura et al. 2013). This number may be affected by two factors. The first is the ability of germ cells to induce mitosis, which varies depending on the chicken breed. For example, chicken breeds such as the White Leghorn, Barred Plymouth Rock, and Fayoumi differ in their ability to accept foreign PGC, with the best results being noted for the first of these breeds and the worst for the Barred Plymouth Rock. The second factor regulating the ratio of the number of host germ cells to artificially introduced cells is the number of exogenous PGC which reach the gonads and colonize them, as well as the number of endogenous PGC already present there. Their relative proportions can be increased by the partial or complete removal of endogenous PGC by sterilization. In order to increase the efficiency of germline transmission and genetic modification, a number of methods of inactivation and removal of endogenous PGC from recipient embryos have been developed. The most important of these include: surgical removal (Naito et al. 1994), inactivation using UV radiation (Reynaud 1976), X-rays (Nakamura et al. 2012) and γ radiation (Carsience et al. 1993), and chemical treatment with busulfan (Nakamura et al. 2009; Lee et al. 2013) and tamoxifen (Mohsen and Ahmed 2002).

Some of these methods are impractical in the routine production of chimeras because they also affect the embryonic development of the recipient (Naito 2003). The mortality of embryos exposed to irradiation combined with PGC injection was higher than that of control embryos and embryos exposed to irradiation (Maeda et al. 1998). Moreover, the same authors demonstrated a reduction in the size of gonads because of the general delay in development induced by γ irradiation. The survival rate of the chick embryos after the removal of a cell cluster from the blastoderm was 59.8% (579 of 969) at day 3 of incubation (Naito et al. 1999).

The sex of donor PGC and recipient PGC is important because the frequency of donor-derived offspring from germline chimeric chickens was significantly higher for the same-sex combinations of donor PGC and recipient embryos when compared with different-sex combinations (reviewed by Naito 2015). When the sex of the donor embryo was the same as that of the recipient embryo, 62.5% to 68.2% of chimeric chickens produced donor-derived offspring. When the sex of the donor embryo differed from that of the recipient embryo, 11.1% to 22.2% of chimeric chickens produced donor-derived offspring (Naito et al. 1999).

Information about the hatchability of manipulated embryos, the raising and identification of germline chimeras, mating germline chimeras, transgene inheritance, and transgene expression is limited. Some of these data are summarized in Table 1.

**Table 1:** Hatchability, germline chimera rates, and transgene inheritance in manipulated chickens

Authors	Number of manipulated embryos	Hatchability, %	Germline chimeras, no. (%)	Transgene inheritance, no.( %)
Naito et al. (1998)			2/18 (11.1)	0 (0)
Naito et al. (1999)	314	26.4		
van de Lavoir et al. (2006)				Yes
Macdonald et al. (2012)	16	18.8*	1/3 (33.3)	1/518 (0.002)
Park and Han (2012a)			228/459 (52.2)	
Tyack et al. (2013)	40	40.0	5/11 (45.5)	5/419 (1.19)
Miyahara et al. (2016)			2/59 (3.4)	

*Survived until sexual maturity

These data indicate that the efficiency of chimera production is still very low. Some data indicate that germline chimeras exhibit significant alterations in sex hormone levels in the ovary and blood plasma, which may affect their reproductive abilities (Sechman et al. 2006). Also, breeding of the chimeras did not often result in germline transmission of the transgene, indicating that the contribution of the transgenic cells to the germline is either non-existent or very low. In this context, the results published by Trefil et al. (2017) are interesting. They obtained viable genetically modified offspring from male PGC matured in the adult testes of sterilized recipient roosters. This new technique eliminates the germline chimerism of G0 roosters and, therefore, it seems to be faster, more efficient, and requires fewer animals.

## Conclusions

The non-viral, cell-based strategy has been used in the generation of transgenic chickens; however, the efficiency of chimera production, transgene inheritance, and transgene expression is still very low. Thus, although this strategy demonstrates that transgenic chickens could successfully produce the humane therapeutic proteins in their oviducts, widespread adoption of this methodology requires additional information. The relationship between embryonic stem cells and germline competency and transgene expression is still an open field of investigation. Presently, very little is known about the effects of primordial germ cells (PGC) manipulation, long-term cultivation in vitro, and different cell/tissue environments in the recipient embryo on the cell phenotypes (morphology, behavior, gene expression, DNA methylation) and on their functional ability. Additionally, more data are required at all cellular, molecular, and biological levels to draw conclusions on the relationship between the reproductive characteristics and physiological state of the chimera, the site genomic integration of the transgene, and the number of its copies, and the level of its expression in the oviduct. Furthermore, the generation of transgenic chickens through chimeric intermediates is a highly skilled and costly process, making it difficult to apply in the general laboratory. From this point of view, it is apparent that the development of simple methods of PGC isolation and genetic modification, and their transfer into recipient embryos, is the essential step for the production of transgenic chickens.
